# 2,6-Dichloro-*N*-(4-chloro­phen­yl)benzamide

**DOI:** 10.1107/S1600536812007556

**Published:** 2012-02-24

**Authors:** Jing Zhu, Ming Li, Hong-xia Wei, Jian-qiang Wang, Cheng Guo

**Affiliations:** a College of Science, Nanjing University of Technology, Xinmofan Road No. 5 Nanjing, Nanjing 210009, People’s Republic of China

## Abstract

In the title compound, C_13_H_8_Cl_3_NO, the dihedral angle between the benzene rings is 63.2 (2)°. In the crystal, N—H⋯O hydrogen bonds link the mol­ecules into *C*(4) chains propagating in [001]. Weak aromatic π–π stacking also occurs [centroid–centroid separations = 3.759 (3) and 3.776 (3) Å].

## Related literature

For further synthetic details, see: Lai & Huang (2005 ▶).
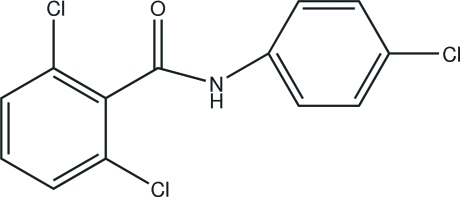


## Experimental

### 

#### Crystal data

C_13_H_8_Cl_3_NO
*M* *_r_* = 300.55
Monoclinic, 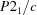

*a* = 11.241 (2) Å
*b* = 12.590 (3) Å
*c* = 9.6450 (19) Å
β = 100.60 (3)°
*V* = 1341.7 (5) Å^3^
*Z* = 4
Mo *K*α radiation
μ = 0.67 mm^−1^
*T* = 293 K
0.30 × 0.20 × 0.10 mm

#### Data collection

Enraf–Nonius CAD-4 diffractometer
Absorption correction: ψ scan (North *et al.*, 1968 ▶) *T*_min_ = 0.825, *T*_max_ = 0.936
2587 measured reflections
2459 independent reflections
1481 reflections with *I* > 2σ(*I*)
*R*_int_ = 0.031
3 standard reflections every 200 reflections intensity decay: 1%

#### Refinement

*R*[*F* ^2^ > 2σ(*F* ^2^)] = 0.057
*wR*(*F* ^2^) = 0.181
*S* = 1.00
2459 reflections
163 parameters
H-atom parameters constrained
Δρ_max_ = 0.26 e Å^−3^
Δρ_min_ = −0.29 e Å^−3^

### 

Data collection: *CAD-4 EXPRESS* (Enraf–Nonius, 1989 ▶); cell refinement: *CAD-4 EXPRESS*; data reduction: *XCAD4* (Harms & Wocadlo, 1995 ▶); program(s) used to solve structure: *SHELXS97* (Sheldrick, 2008 ▶); program(s) used to refine structure: *SHELXL97* (Sheldrick, 2008 ▶); molecular graphics: *SHELXL97*; software used to prepare material for publication: *PLATON* (Spek, 2009 ▶).

## Supplementary Material

Crystal structure: contains datablock(s) global, I. DOI: 10.1107/S1600536812007556/hb6644sup1.cif

Structure factors: contains datablock(s) I. DOI: 10.1107/S1600536812007556/hb6644Isup2.hkl

Supplementary material file. DOI: 10.1107/S1600536812007556/hb6644Isup3.cml

Additional supplementary materials:  crystallographic information; 3D view; checkCIF report

## Figures and Tables

**Table 1:** Hydrogen-bond geometry (Å, °)

*D*—H⋯*A*	*D*—H	H⋯*A*	*D*⋯*A*	*D*—H⋯*A*
N—H0*A*⋯O^i^	0.86	1.97	2.828 (4)	176

Symmetry code: (i) 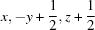.

**Fig. 1.**
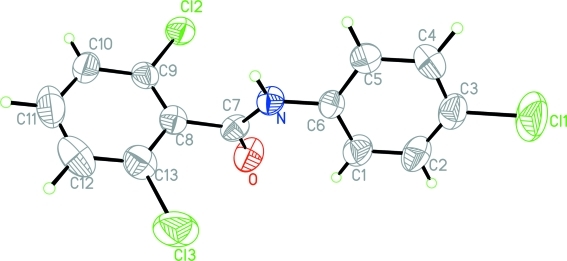
The molecular structure of the title molecule, with displacement ellipsoids drawn at the 50% probability level.

**Fig. 2.**
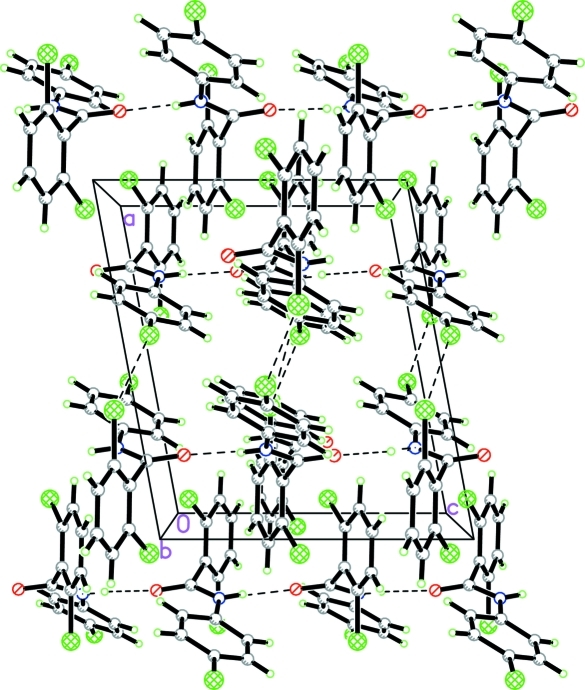
A packing diagram of (I) viewed down the b axis. Hydrogen bonds are drawn as dashed lines.

## supplementary crystallographic information

### Experimental

2,6-Dichlorobenzoyl chloride (0.02 mol, 4.20 g)and 4-chloroaniline (0.02 mol, 2.55 g) were refluxed in triethylamine (6 ml) and tetrahydrofuran (50 ml) for 8h, then the solvents were evaporated to give raw product, which was finally washed by water and collected by filtration. Colourless blocks were obtained by slow evaporation of an ethyl acetate solution.

### Refinement

H atoms were positioned geometrically, with N-H = 0.86 Å and C-H = 0.93 Å for aromatic H, and constrained to ride on their parent atoms, with U_iso_(H) = 1.2U_eq_(C,N).

### Crystal data

**Table:**

C_13_H_8_Cl_3_NO	*D*_x_ = 1.488 Mg m^−^^3^
*M**_r_* = 300.55	Melting point: 397 K
Monoclinic, *P*2_1_/*c*	Mo *K*α radiation, λ = 0.71073 Å
*a* = 11.241 (2) Å	Cell parameters from 25 reflections
*b* = 12.590 (3) Å	θ = 9–13°
*c* = 9.6450 (19) Å	µ = 0.67 mm^−^^1^
β = 100.60 (3)°	*T* = 293 K
*V* = 1341.7 (5) Å^3^	Block, colourless
*Z* = 4	0.30 × 0.20 × 0.10 mm
*F*(000) = 608	

### Data collection

**Table:**

Enraf–Nonius CAD-4 diffractometer	1481 reflections with *I* > 2σ(*I*)
Radiation source: fine-focus sealed tube	*R*_int_ = 0.031
Graphite monochromator	θ_max_ = 25.4°, θ_min_ = 1.8°
ω/2θ scans	*h* = −13→0
Absorption correction: ψ scan (North *et al.*, 1968)	*k* = 0→15
*T*_min_ = 0.825, *T*_max_ = 0.936	*l* = −11→11
2587 measured reflections	3 standard reflections every 200 reflections
2459 independent reflections	intensity decay: 1%

### Refinement

**Table:**

Refinement on *F*^2^	Primary atom site location: structure-invariant direct methods
Least-squares matrix: full	Secondary atom site location: difference Fourier map
*R*[*F*^2^ > 2σ(*F*^2^)] = 0.057	Hydrogen site location: inferred from neighbouring sites
*wR*(*F*^2^) = 0.181	H-atom parameters constrained
*S* = 1.00	*w* = 1/[σ^2^(*F*_o_^2^) + (0.095*P*)^2^] where *P* = (*F*_o_^2^ + 2*F*_c_^2^)/3
2459 reflections	(Δ/σ)_max_ < 0.001
163 parameters	Δρ_max_ = 0.26 e Å^−^^3^
0 restraints	Δρ_min_ = −0.29 e Å^−^^3^

### Special details

**Table:**

Geometry. All esds (except the esd in the dihedral angle between two l.s. planes) are estimated using the full covariance matrix. The cell esds are taken into account individually in the estimation of esds in distances, angles and torsion angles; correlations between esds in cell parameters are only used when they are defined by crystal symmetry. An approximate (isotropic) treatment of cell esds is used for estimating esds involving l.s. planes.
Refinement. Refinement of F^2^ against ALL reflections. The weighted R-factor wR and goodness of fit S are based on F^2^, conventional R-factors R are based on F, with F set to zero for negative F^2^. The threshold expression of F^2^ > 2sigma(F^2^) is used only for calculating R-factors(gt) etc. and is not relevant to the choice of reflections for refinement. R-factors based on F^2^ are statistically about twice as large as those based on F, and R- factors based on ALL data will be even larger.

### Fractional atomic coordinates and isotropic or equivalent isotropic displacement parameters (Å^2^)

**Table:**

	*x*	*y*	*z*	*U*_iso_*/*U*_eq_	
N	0.7580 (3)	0.3117 (2)	0.1127 (3)	0.0423 (8)	
H0A	0.7677	0.2870	0.1972	0.051*	
O	0.7776 (3)	0.2668 (2)	−0.1093 (3)	0.0660 (9)	
Cl1	0.57123 (16)	0.75067 (10)	0.0703 (2)	0.1055 (6)	
C1	0.7243 (4)	0.4801 (4)	−0.0171 (5)	0.0593 (12)	
H1A	0.7651	0.4547	−0.0859	0.071*	
Cl2	0.64198 (10)	0.05573 (10)	0.07481 (12)	0.0617 (4)	
C2	0.6810 (5)	0.5830 (4)	−0.0246 (5)	0.0668 (13)	
H2A	0.6917	0.6268	−0.0990	0.080*	
Cl3	1.06285 (13)	0.25773 (11)	0.06642 (19)	0.0927 (6)	
C3	0.6224 (4)	0.6202 (4)	0.0774 (6)	0.0594 (12)	
C4	0.6040 (4)	0.5568 (4)	0.1854 (6)	0.0660 (13)	
H4A	0.5638	0.5829	0.2543	0.079*	
C5	0.6460 (4)	0.4524 (4)	0.1924 (5)	0.0544 (11)	
H5A	0.6323	0.4082	0.2652	0.065*	
C6	0.7070 (3)	0.4148 (3)	0.0926 (4)	0.0394 (9)	
C7	0.7928 (4)	0.2481 (3)	0.0165 (4)	0.0443 (10)	
C8	0.8579 (4)	0.1497 (3)	0.0788 (4)	0.0434 (10)	
C9	0.7988 (4)	0.0590 (3)	0.1085 (4)	0.0465 (10)	
C10	0.8603 (5)	−0.0302 (4)	0.1654 (5)	0.0595 (12)	
H10A	0.8188	−0.0911	0.1831	0.071*	
C11	0.9838 (5)	−0.0267 (5)	0.1951 (6)	0.0765 (15)	
H11A	1.0264	−0.0859	0.2346	0.092*	
C12	1.0461 (5)	0.0615 (5)	0.1683 (6)	0.0753 (15)	
H12A	1.1302	0.0628	0.1906	0.090*	
C13	0.9836 (4)	0.1479 (4)	0.1082 (5)	0.0593 (12)	

### Atomic displacement parameters (Å^2^)

**Table:**

	*U*^11^	*U*^22^	*U*^33^	*U*^12^	*U*^13^	*U*^23^
N	0.055 (2)	0.0430 (19)	0.0289 (16)	0.0040 (16)	0.0070 (14)	0.0032 (15)
O	0.109 (3)	0.0540 (19)	0.0365 (17)	0.0039 (17)	0.0164 (17)	−0.0042 (14)
Cl1	0.1079 (13)	0.0498 (8)	0.1576 (16)	0.0283 (8)	0.0215 (11)	0.0106 (9)
C1	0.077 (3)	0.052 (3)	0.051 (3)	0.006 (2)	0.018 (2)	0.010 (2)
Cl2	0.0515 (7)	0.0637 (7)	0.0707 (8)	−0.0022 (5)	0.0132 (5)	0.0087 (6)
C2	0.085 (4)	0.046 (3)	0.068 (3)	0.004 (3)	0.011 (3)	0.016 (2)
Cl3	0.0706 (9)	0.0743 (10)	0.1392 (14)	−0.0252 (7)	0.0347 (9)	−0.0239 (9)
C3	0.048 (3)	0.043 (3)	0.084 (4)	0.003 (2)	0.002 (2)	0.003 (2)
C4	0.063 (3)	0.063 (3)	0.078 (3)	0.013 (2)	0.028 (3)	−0.007 (3)
C5	0.062 (3)	0.052 (3)	0.053 (3)	0.004 (2)	0.019 (2)	0.006 (2)
C6	0.042 (2)	0.036 (2)	0.039 (2)	−0.0007 (17)	0.0032 (17)	−0.0009 (17)
C7	0.058 (3)	0.044 (2)	0.031 (2)	−0.0057 (19)	0.0088 (18)	−0.0004 (18)
C8	0.052 (2)	0.043 (2)	0.036 (2)	0.0019 (19)	0.0082 (18)	−0.0073 (18)
C9	0.049 (2)	0.052 (2)	0.040 (2)	0.006 (2)	0.0127 (18)	−0.001 (2)
C10	0.071 (3)	0.049 (3)	0.061 (3)	0.013 (2)	0.021 (2)	0.011 (2)
C11	0.076 (4)	0.076 (4)	0.076 (4)	0.032 (3)	0.008 (3)	0.011 (3)
C12	0.046 (3)	0.095 (4)	0.082 (4)	0.013 (3)	0.003 (3)	−0.013 (3)
C13	0.055 (3)	0.052 (3)	0.071 (3)	−0.003 (2)	0.013 (2)	−0.015 (2)

### Geometric parameters (Å, º)

**Table:**

N—C7	1.338 (5)	C4—H4A	0.9300
N—C6	1.417 (5)	C5—C6	1.366 (5)
N—H0A	0.8600	C5—H5A	0.9300
O—C7	1.216 (4)	C7—C8	1.506 (5)
Cl1—C3	1.737 (5)	C8—C9	1.378 (6)
C1—C6	1.381 (5)	C8—C13	1.389 (6)
C1—C2	1.382 (6)	C9—C10	1.379 (6)
C1—H1A	0.9300	C10—C11	1.365 (7)
Cl2—C9	1.733 (4)	C10—H10A	0.9300
C2—C3	1.365 (7)	C11—C12	1.363 (7)
C2—H2A	0.9300	C11—H11A	0.9300
Cl3—C13	1.732 (5)	C12—C13	1.365 (7)
C3—C4	1.358 (7)	C12—H12A	0.9300
C4—C5	1.394 (6)		
			
C7—N—C6	128.0 (3)	O—C7—N	124.9 (4)
C7—N—H0A	116.0	O—C7—C8	121.7 (4)
C6—N—H0A	116.0	N—C7—C8	113.4 (3)
C6—C1—C2	120.1 (4)	C9—C8—C13	117.1 (4)
C6—C1—H1A	120.0	C9—C8—C7	123.2 (4)
C2—C1—H1A	120.0	C13—C8—C7	119.7 (4)
C3—C2—C1	119.8 (4)	C8—C9—C10	122.2 (4)
C3—C2—H2A	120.1	C8—C9—Cl2	119.6 (3)
C1—C2—H2A	120.1	C10—C9—Cl2	118.3 (3)
C4—C3—C2	120.9 (4)	C11—C10—C9	118.2 (5)
C4—C3—Cl1	119.4 (4)	C11—C10—H10A	120.9
C2—C3—Cl1	119.7 (4)	C9—C10—H10A	120.9
C3—C4—C5	119.6 (4)	C12—C11—C10	121.7 (5)
C3—C4—H4A	120.2	C12—C11—H11A	119.2
C5—C4—H4A	120.2	C10—C11—H11A	119.2
C6—C5—C4	120.2 (4)	C11—C12—C13	119.1 (5)
C6—C5—H5A	119.9	C11—C12—H12A	120.4
C4—C5—H5A	119.9	C13—C12—H12A	120.4
C5—C6—C1	119.5 (4)	C12—C13—C8	121.7 (5)
C5—C6—N	117.6 (3)	C12—C13—Cl3	119.2 (4)
C1—C6—N	122.7 (4)	C8—C13—Cl3	119.1 (4)
			
C6—C1—C2—C3	0.8 (7)	O—C7—C8—C13	82.0 (5)
C1—C2—C3—C4	−1.2 (8)	N—C7—C8—C13	−96.3 (5)
C1—C2—C3—Cl1	178.4 (4)	C13—C8—C9—C10	0.3 (6)
C2—C3—C4—C5	0.2 (7)	C7—C8—C9—C10	179.9 (4)
Cl1—C3—C4—C5	−179.4 (4)	C13—C8—C9—Cl2	179.9 (3)
C3—C4—C5—C6	1.2 (7)	C7—C8—C9—Cl2	−0.5 (5)
C4—C5—C6—C1	−1.6 (6)	C8—C9—C10—C11	1.2 (6)
C4—C5—C6—N	173.8 (4)	Cl2—C9—C10—C11	−178.5 (4)
C2—C1—C6—C5	0.6 (7)	C9—C10—C11—C12	−0.8 (8)
C2—C1—C6—N	−174.5 (4)	C10—C11—C12—C13	−1.1 (8)
C7—N—C6—C5	161.0 (4)	C11—C12—C13—C8	2.6 (8)
C7—N—C6—C1	−23.7 (6)	C11—C12—C13—Cl3	−177.0 (4)
C6—N—C7—O	−5.6 (7)	C9—C8—C13—C12	−2.2 (6)
C6—N—C7—C8	172.6 (3)	C7—C8—C13—C12	178.2 (4)
O—C7—C8—C9	−97.7 (5)	C9—C8—C13—Cl3	177.4 (3)
N—C7—C8—C9	84.1 (5)	C7—C8—C13—Cl3	−2.2 (5)

### Hydrogen-bond geometry (Å, º)

**Table:**

*D*—H···*A*	*D*—H	H···*A*	*D*···*A*	*D*—H···*A*
N—H0*A*···O^i^	0.86	1.97	2.828 (4)	176

Symmetry code: (i) *x*, −*y*+1/2, *z*+1/2.
